# Frontal Sinus “Hump”: An Anatomical Risk Factor for Anterior Skull Base Injury in the Endoscopic Modified Lothrop Approach (Outside-In Frontal Drill-Out)

**DOI:** 10.1155/2021/3402496

**Published:** 2021-07-22

**Authors:** Teppei Takeda, Kazuhiro Omura, Haw Torng, Teru Ebihara, Satoshi Aoki, Kosuke Tochigi, Hiromi Kojima, Nobuyoshi Otori

**Affiliations:** ^1^Department of Otorhinolaryngology, The Jikei University School of Medicine, 3-25-8 Nishi-shinbashi, Minato-ku, Tokyo 105-8461, Japan; ^2^Department of Otorhinolaryngology, Dokkyo Medical University Saitama Medical Center, 2-1-50 Minami-koshigaya, Koshigaya-Shi, Saitama 350-8550, Japan

## Abstract

Skull base injuries caused by the outside-in frontal drill-out technique have not been reported. In this report, we chose an outside-in approach to open the frontal sinus for olfactory neuroblastoma resection. Although we identified the first olfactory fibre, the anterior skull base was damaged while drilling into the frontal sinus on the tumour side. We reconstructed the skull base in multiple layers using fascia and cartilage. Postoperative cerebrospinal fluid leakage or intracranial haemorrhage was not observed. In this case, a morphological difference existed in the posterior wall of the frontal sinus between the right and left sides, like a “hump” in the posterior wall of the frontal sinus. This case of damage to the anterior skull base that could not be avoided by identifying the first olfactory fibre alone is the first published case of skull base injury caused by the outside-in approach due to morphological variations of the frontal sinus and skull base. In this approach, the posterior wall of the frontal sinus cannot be observed because the intraoperative landmark is limited to the first olfactory fibre. Therefore, morphological variations of the posterior wall of the frontal sinus should be analysed in advance to prevent cranial base injury.

## 1. Introduction

The history of extended approaches to the frontal sinus begins from the approach proposed by Lothrop [1, 2]. Draf, Close, and Gross each published a modified version of the procedure in the 1990s [3–5]. The method later became known as the endoscopic modified Lothrop procedure (EMLP) or Draf III procedure [3]. Currently, this procedure is indicated for cases of persistent chronic sinusitis with inadequate response to medical therapy, primary frontal sinus surgery failure, frontal sinus mucoceles and tumours, frontoethmoid fractures, and endoscopic skull base surgery [3, 6].

Knisely et al. reported an “outside-in” frontal drill-out approach as an EMLP that allows early surgical orientation and fast bone removal [7, 8]. This method uses the first olfactory fibre, which is not easily distorted by disease, as the posterior limit of the frontal sinus, and uses it as a landmark to drill out the upper nasofrontal beak; the frontal sinus can be safely opened in this way without damaging the anterior cranial base [7, 8]. Ipsilateral access to the frontal sinus recess can be hindered by several circumstances, including the presence of a tumour, scarring, outflow tract osteogenesis, or fat prolapse from previous medial orbital wall decompression or trauma [9]; the outside-in approach is a good adaptation in these cases. The major complications of the EMLP include cerebrospinal fluid (CSF) leakage and posterior table dehiscence, which have been reported to occur in <1% of patients [10]; however, there have been no reports of skull base injuries caused by the outside-in approach.

In this case, we performed an endoscopic anterior cranial resection of an olfactory neuroblastoma and chose the outside-in approach to open the frontal sinus. Although the first olfactory fibre was identified, the anterior skull base was damaged during drilling into the frontal sinus on the tumour side. This case of damage to the anterior skull base that could not be avoided by identifying the first olfactory fibre alone is the first published case of skull base injury caused by the outside-in frontal drill-out technique due to morphological variations of the frontal sinus and skull base.

## 2. Case Report/Case Presentation

This report is based on the informed consent of the patient and the approval of the appropriate ethics committee.

**Figure 1:**
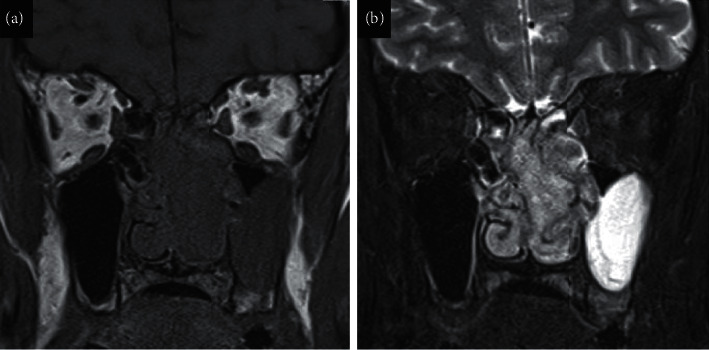
Magnetic resonance imaging. (a) T1-weighted image on coronal section. (b) T2-weighted image on coronal section.

**Figure 2:**
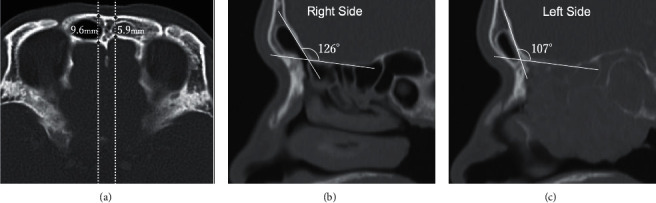
Computed tomography of the frontal sinus. (a) Axial section. The anteroposterior (AP) diameter of the right frontal sinus is 9.6 mm and that of the left frontal sinus is 5.9 mm. (b, c) Sagittal section of the right and left frontal sinuses. The skull base-frontal sinus angle (SBA) on the right frontal sinus is 126° and that of the left frontal sinus is 107°.

We report a case of a 56-year-old man with an olfactory neuroblastoma. The patient's chief complaint was nasal obstruction and left epistaxis. Magnetic resonance imaging showed that the tumour occupied mainly the left olfactory cleft and was isointense on T1 and high intense on T2 and showed no thickening of the left dura mater or invasion of the middle cranial fossa (shown in Figures 1(a) and 1(b)). The left frontal sinus was filled with secondary mucus, and no tumour component was observed. However, both sides of the frontal sinuses were hypoplastic, with thickening of the septum of the frontal sinus and a difference in the anteroposterior (AP) diameter of the frontal sinus between the right and left sides (shown in Figure 2(a)). The AP diameter was 9.6 mm on the right and 5.9 mm on the left, and the skull base-frontal sinus angle (SBA) was 126° on the right and 107° on the left (shown in Figures 2(b) and 2(c)).

**Figure 3:**
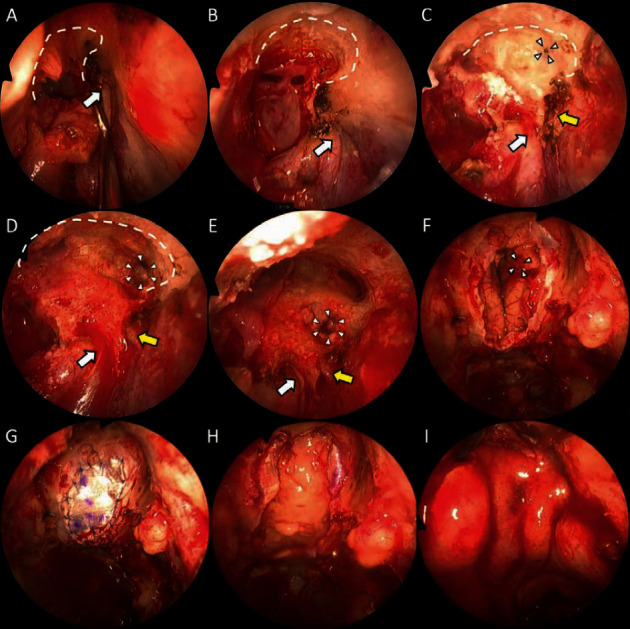
Surgical image presentation. (a) The endoscopic modified Lothrop procedure (EMLP) on the right side. The first olfactory fibre was identified (white arrow), and the right frontal beak was drilled out (white dotted line). (b) The posterior wall of the right frontal sinus was identified, the floor of the frontal sinus (white dotted line) was drilled out, and the left frontal sinus was approached. The first olfactory fibre is visible (white arrow). (c) After drilling out the floor of the frontal sinus. The first olfactory fibre on the left side (tumour side) is identified (yellow arrow), and a space was observed (white triangle) on the left side at the same depth as the right frontal sinus. (d) The space was drilled out to open by 4 mm (white triangle); leakage of cerebrospinal fluid was observed. The first olfactory fibre (white and yellow arrow). (e): The EMLP was finished. Bilateral frontal sinuses were observed; it was confirmed that the posterior wall of the left frontal sinus was damaged (white triangle). The first olfactory fibre (white and yellow arrow). (f) After anterior cranial resection of an olfactory neuroblastoma. The injured area of the skull base overlapped with the resected area of the tumour. (g) The skull base was reconstructed to be water-tight by suturing the dura mater to the fascia. (h) Multilayer reconstruction using fascia and cartilage. (i) Finally, the wound was covered with a nasoseptal flap.

The patient underwent endoscopic anterior cranial resection for treatment. To determine the anterior tumour resection line, the outside-in EMLP was selected. First, the first olfactory fibre was identified on the right side (non-tumour side) (shown in Figure 3), and the depths of the right frontal sinus and the right anterior skull base were confirmed by Draf 2B (Figure 3). Subsequently, Draf 2D [9] was used to approach the left frontal sinus from the right frontal sinus via the septum of the frontal sinus (shown in Figure 3). At this time, the first olfactory fibre was identified on the left side (tumour side), and a space was observed on the left side at the same depth as the right frontal sinus, and the space was opened as if it was the frontal sinus. After opening the space by 4 mm, CSF leakage was observed, and the skull base was judged to be damaged (shown in Figure 3). When the position of the frontal sinuses was reoriented and the bilateral frontal sinuses were converted to a single sinus, the left anterior skull base protruded more than the right anterior skull base, and it was confirmed that the protruded region was damaged (shown in Figure 3). Fortunately, the injured area of the skull base overlapped with the resected area of the tumour (shown in Figure 3), and the skull base was reconstructed to be water-tight by suturing the dura mater to the fascia with 11 stitches of 6-0 proline (shown in Figure 3). Additionally, the fascia and nasal septal cartilage were in-layed between the dura mater and the skull base (shown in Figure 3). Finally, the nasoseptal flap was over-layed to complete the operation (shown in Figure 3). The postoperative course was good without CSF leakage or intracranial haemorrhage, and the patient was discharged one week later. There was no recurrence or higher-order functional disability observed 1 year after surgery.

## 3. Discussion/Conclusion

We reported a case of damage to the anterior skull base that could not be avoided by identifying the first olfactory fibre alone. This is the first published case of skull base injury caused by the outside-in frontal drill-out EMLP due to morphological variations of the frontal sinus and skull base.

**Figure 4:**
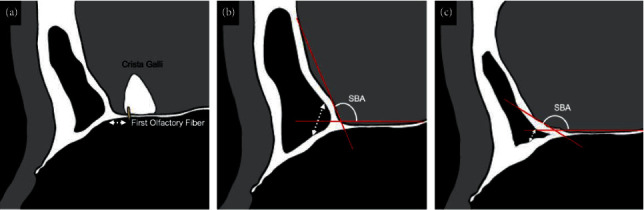
Relationship between the floor of the frontal sinus, the posterior wall of the frontal sinus, and the first olfactory fibre in sagittal computed tomography. (a) Relationship between the first olfactory fibre and the posterior wall of the frontal sinus. (b, c) Relationship between the skull base-frontal sinus angle (SBA) and the posterior wall of the frontal sinus.

It is necessary to identify the anatomical landmarks intraoperatively to prevent skull base injury during EMLP. Furthermore, the anatomical landmarks are the first olfactory fibre, the bilateral frontal sinuses, and the anterior skull base in the inside-out approach. In contrast, in the outside-in drill-out approach, only the first olfactory fibre, which is the posterior limit of the frontal sinuses, is the landmark, and the endoscopic findings in the same field of view are less landmarked than in the inside-out approach. However, Knisely et al. reported that the strong adherence of the first olfactory fibre through the periosteal sheath as it enters the cribriform facilitates surgical orientation as an absolutely fixed landmark without distortion due to individual variation or disease [7]. Furthermore, Upadhyay et al. investigated the relationship between the first olfactory fibre and the posterior wall of the frontal sinus using 15 cadaveric specimens to evaluate whether the first olfactory fibre can be used as a surgical landmark for the posterior wall of the frontal sinus [11]. Reportedly, the posterior wall of the frontal sinus is on average 4 mm upward from the first olfactory fibre in the tangential direction of the endoscope, and the distance between the first olfactory fibre and the posterior wall of the frontal sinus increases as the AP diameter increases [11]. Drilling no further posterior than 7 mm rostral to the first olfactory fibre would be safe in 91% of patients [11]. In other words, the first olfactory fibre is used as an anatomical landmark to avoid inadvertently damaging the anterior skull base posterior to the frontal sinus when drilling out the floor of the frontal sinus (shown in Figure 4(a)). In this case, the anterior skull base was injured during the outside-in EMLP even though all the above precautions were taken. Therefore, to prevent this injury, it is necessary to consider other anatomical factors in addition to identifying the first olfactory fibre alone.

In this case, the posterior wall of the frontal sinus within the frontal sinus was injured. It is necessary to confirm the morphological variation of the anterior skull base by preoperative computed tomography to prevent this skull base injury. When observing the morphological characteristics of the anterior skull base, there is a method of evaluating the SBA [11], which we focused on. This evaluates the angle of the posterior wall of the frontal sinus, and as the SBA becomes acute, the posterior wall of the frontal sinus approaches the floor of the frontal sinus (shown in Figures 4(b) and 4(c)). In other words, the more acute the SBA is, the more likely it is that the posterior wall of the frontal sinus will be damaged during the drill-out of the floor of the frontal sinus. There have been no reports on the difference in the SBA between the right and left sides or on the relationship between the SBA and the AP diameter. In this case, the SBA was 126° on the right side and 107° on the left side, indicating a difference in the posterior wall of the frontal sinus between the right and left sides. This difference of 19° in the SBA made it appear as if there was a bony protruding “hump” in the left anterior skull base. Additionally, the AP diameter was narrow, and the septum of the frontal sinus was thickened, which made it difficult to identify the left frontal sinus. We believe that the large right SBA on the normal side, or landmarking the side with the longer distance from the floor of the frontal sinus to the posterior wall of the frontal sinus and drilling out to the opposite side with the shorter distance from the floor of the frontal sinus to the posterior wall of the frontal sinus, led to the injury of the left anterior skull base.

Another reason for the anterior skull base injury is that the axis of the visual field may have been rotated under endoscopy. In the outside-in approach, the landmarks in the surgical field are limited to the first olfactory fibre. As the number of landmarks decreases, the cranial-caudal and lateral orientations become insufficient, which may lead to disorientation of the surgical field, making surgery dangerous. Wormald recommends the use of anatomical landmarks in combination with image guidance to remove the bone above the olfactory fossa [12]. If navigation devices are not available, at least two landmarks should be used in addition to the first olfactory fibre, such as the skin of the nasoorbital area and the heads of the middle turbinates.

In this study, we describe the first skull base injury by outside-in EMLP reported in the scientific literature. Since there are few intraoperative anatomical landmarks in the outside-in approach, preoperative morphological evaluation of the frontal sinus and anterior skull base is necessary, especially in cases of a hypoplastic frontal sinus.

## Data Availability

The data supporting the findings of this study are available from the corresponding author upon request. The data are not publicly available due to privacy and ethical restrictions.

## Supplementary Materials

Supplementary Materials The surgical video of the case with anterior skull base injury can be accessed from the following link: https://youtu.be/iE8vQPfaI-s.
