# Supplementary material for: Frontal Sinus “Hump”: An Anatomical Risk Factor for Anterior Skull Base Injury in the Endoscopic Modified Lothrop Approach (Outside-In Frontal Drill-Out)
Source: Case Rep Otolaryngol. 2021 Jul 22;2021:3402496. doi: 10.1155/2021/3402496 (PMC8321740; doi:10.1155/2021/3402496)
Supplement: Supplementary Materials — The surgical video of the case with anterior skull base injury can be accessed from the following link: https://youtu.be/iE8vQPfaI-s. [file 3402496.f1.docx]

**Supplementary Materials**

Video clips: We provide the surgical video of the case with anterior skull base injury.

You can watch it at the following link.

<https://youtu.be/iE8vQPfaI-s>
